# Supplementary material for: Spin Solid versus Magnetic Charge Ordered State in Artificial Honeycomb Lattice of Connected Elements
Source: Adv Sci (Weinh). 2018 Jan 4;5(4):1700856. doi: 10.1002/advs.201700856 (PMC5908362; doi:10.1002/advs.201700856)
Supplement: Supplementary file 1 — Supplementary [file ADVS-5-1700856-s001.pdf]

Copyright WILEY-VCH Verlag GmbH & Co. KGaA, 69469 Weinheim, Germany, 2018.

ADVANCED SCIENCE
Open Access

Supporting Information

for *Adv. Sci.,* DOI: 10.1002/advs.201700856

Spin Solid versus Magnetic Charge Ordered State in Artificial Honeycomb Lattice of Connected Elements

*Artur Glavic, Brock Summers, Ashutosh Dahal, Joseph Kline, Walter Van Herck, Alexander Sukhov, Arthur Ernst, and Deepak K. Singh**

Supporting Information: Spin Solid versus Magnetic Charge Ordered State in Artificial Honeycomb Lattice of Connected Elements

A. Glavic[1], B. Summers[2], A. Dahal[2], R. J. Kline[3], W. Van Herck[4], A. Sukhov[5], A. Ernst[6,7], and D. K. Singh[2,*]
[1] *Laboratory for Neutron Scattering and Imaging, Paul Scherrer Institut, Villigen PSI, Switzerland*
[2] *Department of Physics and Astronomy, University of Missouri, Columbia, MO 65211*
[3] *National Institute of Standards and Technology, MD 20899*
[4] *Jülich Centre for Neutron Science (JCNS) at Heinz Maier-Leibnitz Zentrum (MLZ), Forschungszentrum Jülich GmbH, Lichtenbergstr. 1, 85748 Garching, Germany*
[5] *Forschungszentrum Jülich GmbH, Helmholtz Institute Erlangen-Nürnberg for Renewable Energy (IEK-11), Fürther Straße 248, 90429 Nürnberg, Germany*
[6] *Institut für Theoretische Physik, Johannes Kepler Universität, A 4040 Linz, Austria*
[7] *Max-Planck-Institut für Mikrostrukturphysik, Weinberg 2, 06120 Halle, Germany and*
[*] *email: singhdk@missouri.edu*

Scattering analysis

Specular reflectivity

Fig. S1 shows the specular reflectivity measured at 300 K together with the model fit from GenX. This measurement was used as a basis to build the structural model for DWBA.

Extraction of GISANS cuts

Two experimental GISANS patterns are shown in Fig. S2 together with the rectangular region that was used to calculate the integrated cuts shown in Fig. 4. Measurement of the Q_y direction is the distinction between doing off-specular scattering and GISANS. For the reflectometry and off-specular measurements, the beam is very divergent in vertical direction, which is the Q_y direction where no resolution is necessary. For GISANS, on the other hand, measurements are performed with significantly reduced vertical collimation slits (the two slits reduced from 30 mm

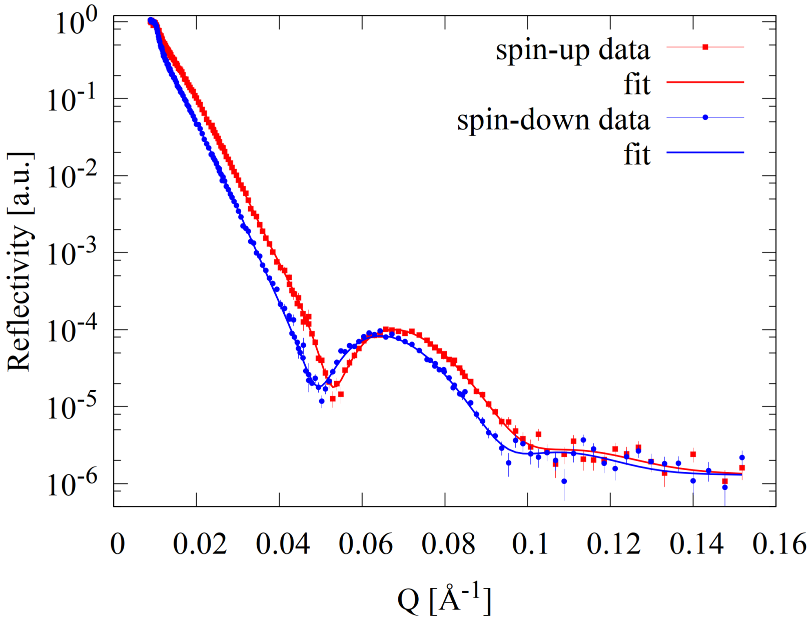

FIG. S1: (Color online) Specular reflectometry data at T = 300 K for spin up and down channels, obtained on an artificial honeycomb lattice. Experimental data is fitted and used as a basis for the DWBA simulation.

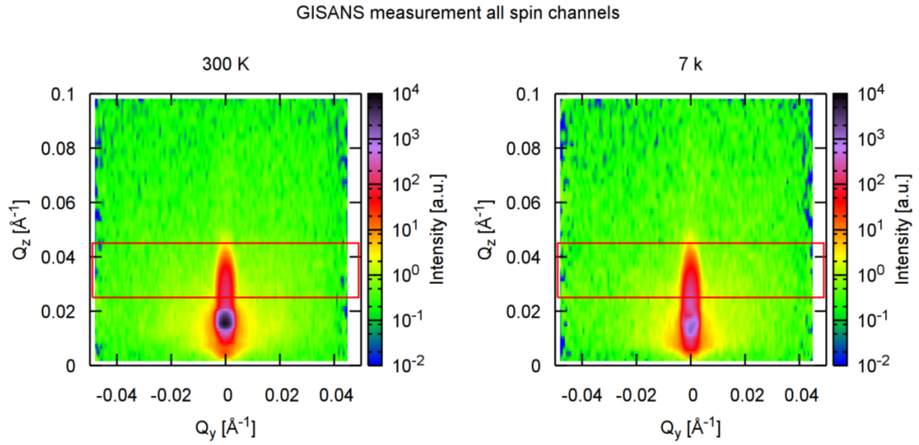

FIG. S2: (Color online) GISANS patterns for all channels at T = 300 K and T = 7 K, respectively.

and 20 mm to ≃ 2 mm and 2 mm).[1] It allowed us to resolve the Q_y direction with the detector, but sacrificed a large amount of intensity. As can be seen, even after combining the full wavelength band and both spin-states, the statistics of the measurements is limited and there is considerable background. Therefore the integrated intensity was used for comparison with the model. The asymmetry between positive and negative Q_y direction is due to instrumental background and likely caused by the instrument geometry, where the negative side is at the bottom of the detector which is partially shielded by the detector arm frame against environmental background.

We did perform GISANS with polarization analysis as well, but the measured signal was not sufficient to be conclusively analyzed.

DWBA modeling

For all simulated intensity patterns shown in the main text and Fig. S3-S5 we have used the same BornAgain sample definition, which was then simulated with different instrument parameters and radiation dependent scattering length density (SLD) values. While the used version of BornAgain did have limitations that prevented to fully reach agreement with the experiments, the modeling allows qualitative comparison to the data and between different magnetic models. (Most significant being no roughness for magnetic models, no interference between structures in different layers.)

The model was build from the substrate and 3 layers to account for the SLD variation extracted from the specular signal. The bottom layer is the region of silicon that is structured by the pattering process, the second layer the structured permalloy with the magnetic phase and the top layer a thin region of different SLD due to partially oxidized material, residual organic pattern and rounded edges of the structure. In the permalloy layer we introduced a hexagonal lattice with a=31 nm out of cylindrical cut-outs with a cylinder radius off 11.2 nm. To be able to later introduce the magnetic phases, we have tripled the unit cell of that structure. The natural imperfections of the self assembled system was implemented by using the 2D paracrystal interference function with a damping (coherence) length of 250 nm. This model was then simulated for all rotations of the crystal lattice in 5° steps and summed together.

The magnetic order was modeled by adding to the structural lattice a set of rectangular magnetized regions on the honeycomb edges with fixed magnetization but different orientation. For the spin solid state these were part of the hexagonal lattice and fixed to the structural lattice to model the long range correlations, while for the other situations all possible combinations of correlated particles (spin-clusters of 1/3/6 spins) were added to the layer separately, without inter-cluster interference.This way all models contain the same amount of spins but different spin-spin correlations.

AFM analysis

In Fig. S6, we have presented the image analysis from atomic force micrograph. As we can see in this figure, the roughness in the thickness of the lattice is about ≃ 0.5 nm on the average.

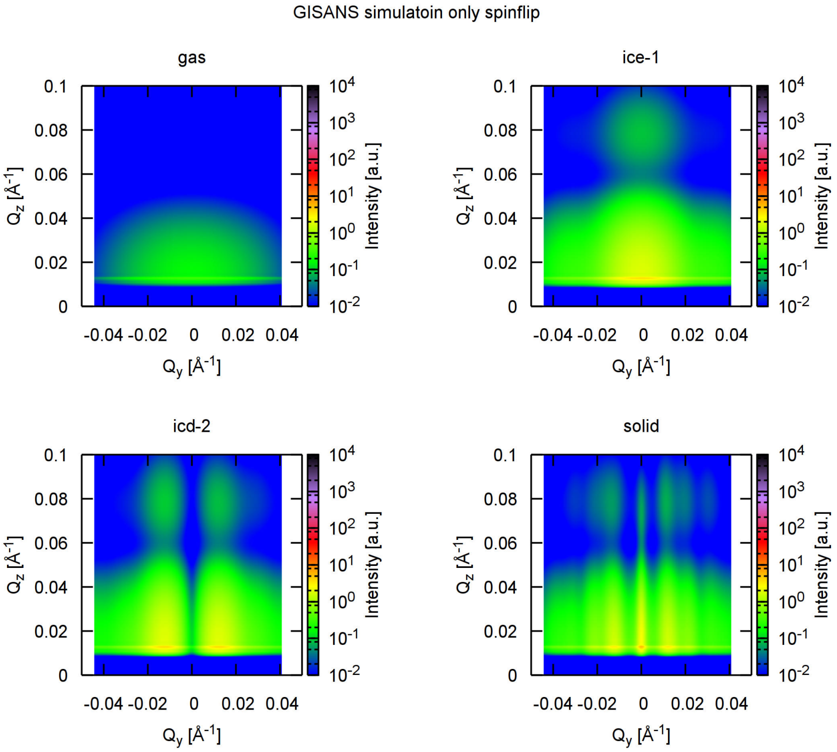

FIG. S3: (Color online) GISANS simulated patterns for the spin flip channel for various magnetic phases in the artificial honeycomb lattice.

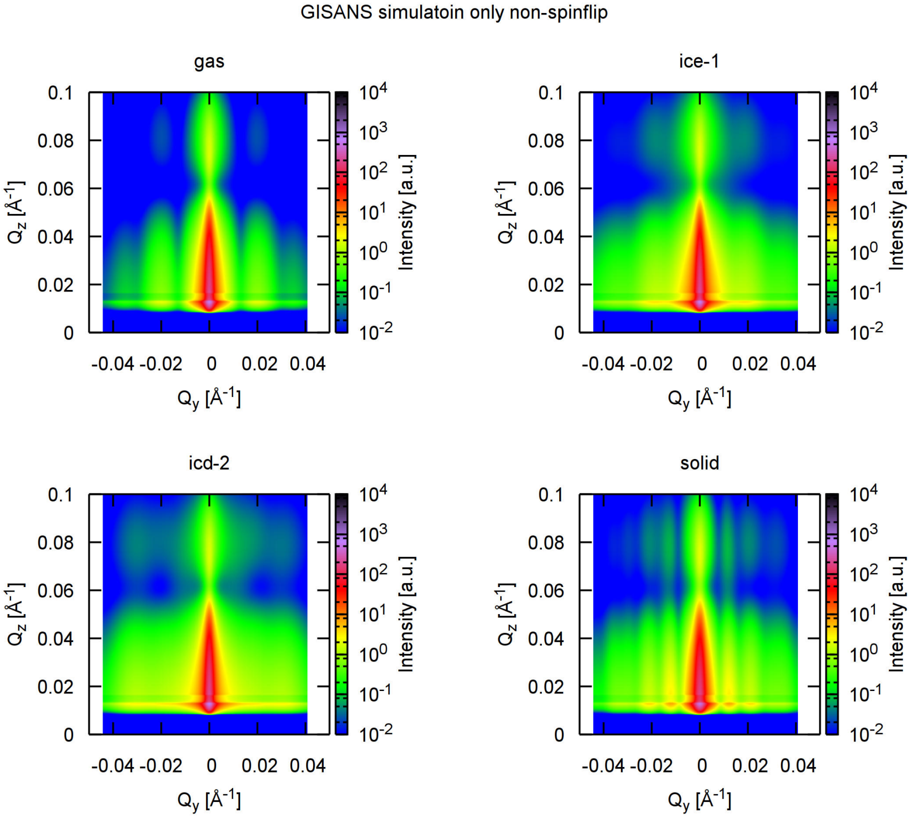

FIG. S4: (Color online) GISANS simulated patterns for the non-spin flip channel for various magnetic phases in the artificial honeycomb lattice.

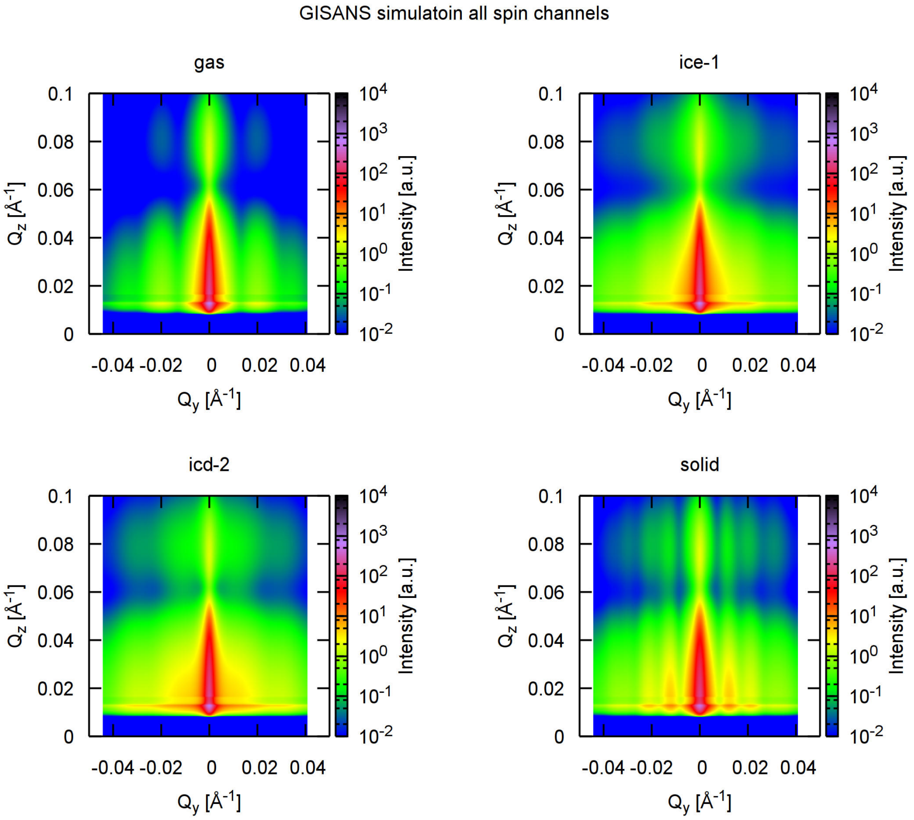

FIG. S5: (Color online) GISANS simulated patterns for all channels for various magnetic phases in the artificial honeycomb lattice.

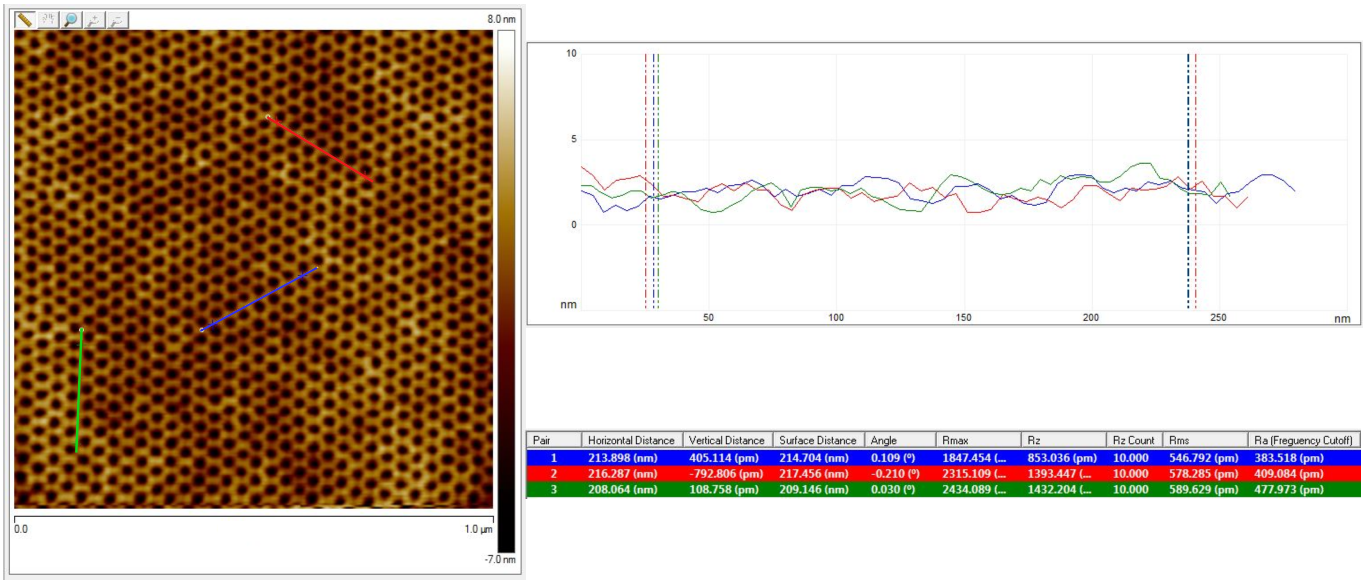

Pair	Horizontal Distance	Vertical Distance	Surface Distance	Angle	Rmax	Rz	Rz Count	Rms	Ra (Fregeuncy Cutoff)
1	213.898 (nm)	405.114 (pm)	214.704 (nm)	0.109 (°)	1847.454 (...	853.036 (pm)	10.000	546.792 (pm)	383.518 (pm)
2	216.287 (nm)	-792.806 (pm)	217.456 (nm)	-0.210 (°)	2315.109 (...	1393.447 (...	10.000	578.285 (pm)	409.084 (pm)
3	208.064 (nm)	108.758 (pm)	209.146 (nm)	0.030 (°)	2434.089 (...	1432.204 (...	10.000	589.629 (pm)	477.973 (pm)

FIG. S6: (Color online) Roughness analysis plot of atomic force micrograph. As shown in the RMS column at the bottom of the plots, the roughness remains somewhere near $\simeq 0.5$ nm across any travel direction along connecting honeycomb elements.

[1] V. Lauter, H.J.C. Lauter, A.Glavic and B.P. Toperverg, *Reference Module in Materials Science and Materials Engineering* **2016**
